# Ovarian ROS-dependent IgG accumulation precedes lipofuscin deposition and follicular decline: comparative insights from the bitch and mouse models of ovarian aging

**DOI:** 10.3389/fragi.2025.1567909

**Published:** 2025-05-30

**Authors:** Luís Montenegro, Natália Rigos, Catarina Brandão, Anabela Pinto, Inês Borges, Luís Cardoso, Hugo Carvalho, Henrique Almeida, Ana Martins-Bessa, Elisabete Silva

**Affiliations:** ^1^ Department of Veterinary Sciences, Universidade de Trás-os-Montes e Alto Douro (UTAD), Vila Real, Portugal; ^2^ Department of Gyneacology and Obstetrics, Veterinary Hospital Referência Veterinária Montenegro, Porto, Portugal; ^3^ Animal and Veterinary Research Center (CECAV), Universidade de Trás-os-Montes e Alto Douro (UTAD), Vila Real, Portugal; ^4^ Associate Laboratory for Animal and Veterinary Sciences (AL4Animals), Lisbon, Portugal; ^5^ Unidade de Biologia Experimental, Departamento de Biomedicina, Faculdade de Medicina, Universidade do Porto, Porto, Portugal; ^6^ Instituto de Investigação e Inovação em Saúde (i3S), Universidade do Porto, Porto, Portugal; ^7^ Instituto de Ciências Biomédicas Abel Salazar, Universidade do Porto, Porto, Portugal; ^8^ Cedivet, Lionesa Business Hub, Matosinhos, Portugal; ^9^ Department of Gyneacology and Obstetrics, Hospital CUF, Porto, Portugal; ^10^ Faculty of Veterinary Medicine, Lisbon University Center, Lusófona University, Lisbon, Portugal; ^11^ IPLUSO – Polytechnic Institute of Lusofonia, School of Health, Protection and Animal Welfare, Lisbon, Portugal

**Keywords:** Ovarian aging, multinucleated giant cell (MNGC), lipofuscin accumulation, IgG, reactive oxygen species

## Abstract

**Introduction:**

In the ovaries, inflammation, oxidative stress, fibrosis and a unique population of multinucleated giant cells have been linked to aging. However, the role of IgG deposition is unknown. Using the dog to study aging is relevant as bitches experience age-related fertility loss and share similar environmental conditions with humans. Therefore, the bitch was used to study reproductive aging. The present work hypothesized that the deposition of multinucleated giant cells and the accumulation of IgGs in the ovary contribute to aging. The objectives were to identify these markers in the ovaries of bitches and correlate them with aging, and to assess whether antioxidants could modulate age-dependent IgG accumulation.

**Methods:**

Ovaries from bitches (from 6 months to 13 years, divided into three groups: <2 years, 2–6 years, and >6 years) and from mice [aged 8–12 weeks–young and 38–42 weeks–reproductively aged (vehicle or apocynin treated)] were employed. Hematoxylin and eosin staining was used to evaluate the ovarian follicle reserve pool. Sudan Black B (SBB) staining identified and characterized the accumulation of lipofuscin, a marker present in ovarian multinucleated giant cells. Immunohistochemistry was employed to determine IgG deposition and western blotting for its quantification. The Kruskal-Wallis and Mann-Whitney-U tests were used for multiple comparisons. The Spearman correlation coefficient measured correlations between the studied variables.

**Results:**

In the bitch, reproductive aging associates with a decrease in follicle pool, an increase in multinucleated giant cells, and an increase in IgG accumulation. Ovarian deposition of lipofuscin was significantly higher in bitches over 2 years of age, whereas IgG deposition was only significant in the >6 years group. Unlike SBB staining, which was absent in the <2 years group, IgG accumulation was already detected in younger animals. In the mice, ovarian IgG staining was increased in reproductively aged animals, but not in reproductively aged animals treated with apocynin.

**Conclusion:**

This study indicates that IgG deposition is an early event that precedes and possibly triggers the recruitment of macrophages. These findings provide new insights into mechanisms of ovarian aging and the use of antioxidants as a strategy to mitigate it.

## 1 Introduction

The average lifespan of humans, domestic species such as dogs and cats, and animals kept in captivity has been consistently extending. For females, many studies indicate that at present a large number of women will live beyond the age of 75 (Baum et al., 2021). In small animals it is common to find bitches over 12–13 years old and queens over 11–12 years old (Montoya et al., 2023). However, this significant increase in lifespan has not been paralleled by a similar increase in female reproductive longevity, which remains practically unchanged and the same as in previous generations. In women, a significant decrease in reproductive capacity occurs around 38 years old (Hawkes and Smith, 2010), in bitches at 7 years (Borge et al., 2011; Mutembei et al., 2002), and in queens at 5 years (Johnson, 2022). This similarity between species regarding age-related loss of fertility makes it possible to study reproductive aging in species other than humans and obtain valuable insights into the mechanisms behind its progression, as evidenced by multiple studies on ovarian and uterine aging in mice and rats. Recently, there has been a growing focus on analyzing aging mechanisms in dogs, not only because they are evolutionarily closer to humans, but also because they are exposed to similar environmental factors, as they share living spaces with us (McCune and Promislow, 2021; Ruple et al., 2022).

Resembling humans, bitches have a reproductive cycle with a constant interestrus period (Concannon, 2011), in contrast to mice, commonly used in studies of female reproductive aging, whose cycle can be induced and accelerated by exposure to males or male urinary scents (Wölfl et al., 2023). Bitches have a monoestrous cycle, with their reproductive life beginning between 5 and 24 months of age, depending on their size and weight. The interestrus period is typically constant for each individual, ranging from 5 to 12 months (Concannon, 2011).

Reproductive aging leads to increased difficulty in achieving pregnancy, a higher incidence of pregnancy-related complications, and ultimately infertility (Ra et al., 2023). The uterus and the ovaries contribute to reproductive dysfunction, but in humans, ovarian aging is a key factor due to the age-related decline in oocyte quality (Ra et al., 2023). In women in their fifth decade, the inability to conceive is more often related to a higher proportion of poor-quality oocytes than to a lack of oocytes (Chen et al., 2022; May-Panloup et al., 2016; te Velde and Pearson, 2002). Gaining a deeper understanding of ovarian aging will be crucial for a deeper understanding of the aging process of the reproductive system and the organism, as the reproductive tract is one of the first systems to show signs of aging.

Studies in mice, goats, and sheep have shown that during ovarian aging the decrease in follicle number is preceded by an increase in the ovarian deposition of multinucleated macrophages containing lipofuscin inclusions (Ansere et al., 2021; Briley et al., 2016; Montenegro et al., 2023; Timóteo-Ferreira et al., 2019), a feature considered a hallmark of ovarian aging (Foley et al., 2021). Additionally, this hallmark may play a role in the decline of oocyte quality and ovarian function. Other changes associated with ovarian aging include increased inflammation, oxidative stress (OS), and fibrosis (Timóteo-Ferreira et al., 2019; Isola et al., 2024; Ra et al., 2023). Antioxidant molecules have been shown to have beneficial effects on reproduction by reducing OS and fibrosis and improving uterine and ovarian function (Gou et al., 2023; Hu et al., 2024; Liu et al., 2013; Shang et al., 2024; Silva et al., 2015; Timóteo-Ferreira et al., 2019). In terms of inflammation and immunity, a recent study (Yu et al., 2024) emphasized the fundamental role of IgG deposition to adipose tissue dysfunction and aging. However, it remains unclear whether IgG deposition also contributes to the processes leading to ovarian aging.

In the present work, it was hypothesized that ovarian deposition of multinucleated giant cells and the accumulation of IgG contribute to its aging. Therefore, the objectives were to identify these markers in the ovaries of bitches and correlate them with aging. Additionally, using a mouse model, the study also aimed to assess whether antioxidants could modulate age-dependent IgG accumulation.

Studying reproductive aging in the bitch may provide valuable insights into human reproductive aging as well. Furthermore, improving female reproductive health can enhance overall wellbeing, leading not only to increased life expectancy but also to a better quality of life for both humans and companion animals.

## 2 Methods

### 2.1 Ovarian tissue collection

Ovaries from bitches aged between 6 months and 13 years were used in the present study. Tissues have been collected from 2021 to 2023 at Veterinary Hospital Referência Veterinária Montenegro (authorized by UTAD ethics committee - Doc43-CE-UTAD-2021). The ovaries were collected as part of an elective ovariohysterectomy procedure. The total number of bitches included in the study was 66. Samples were organized into age groups (n = 3): juvenile group–animals under 2 years old; mature group–animals between 2 and 6 years old inclusive; and reproductively aged animals–animals over 6 years old.

Surgical procedures were conducted under a strict aseptic protocol, allowing the omission of prophylactic antibiotics. The anesthetic protocol included sedation and analgesia with dexmedetomidine and methadone, induction with propofol, and maintenance anesthesia with 2% isoflurane in controlled volatile diffusion. All animals received intravenous fluid support and were intubated to ensure assisted ventilation. The gonadectomy surgical technique, ventromedial laparotomy or laparoscopy, was selected according to the owner’s preference.

Concerning the mouse model of ovarian aging, the experimental design and all procedures were previously conducted and published (Timóteo-Ferreira et al., 2019). A brief description is included below.

Female mice (C57BL/6J strain) obtained from Harlan were kept under controlled conditions (12 h light/dark cycle and room temperature at 22°C) and had free access to tap water and standard mouse chow. Young (8–12 weeks old) and reproductively aged (38–42 weeks old) female mice were employed. To evaluate the possibility of delaying ovarian aging mice were treated with an antioxidant molecule. Reproductively aged mice were divided into two groups, with one receiving apocynin (NADPH-oxidase inhibitor, 5 mM, in drinking water for 7 weeks (Timóteo-Ferreira et al., 2019). At the end of the study, female mice were anesthetized with isoflurane, euthanized and the ovaries were excised. One ovary was frozen in liquid nitrogen and kept at −80°C. The other ovary was embedded in paraffin (using the protocol described below).

All experiments, supervised by a veterinary doctor, were conducted in 2011 in compliance with Portuguese animal welfare legislation and the guidelines issued by the Federation of European Laboratory Animal Science Associations (FELASA).

### 2.2 Paraffin embedding of ovaries and tissue microtomy

Ovaries were fixed in 4% buffered formaldehyde for 24 h and sliced in half according to the longitudinal axis. Tissue was then dehydrated with aid of increasing concentrations of ethanol (70%, 90% and 100%), diaphanized using xylol and embedded in paraffin. Sequential slices (5 µm thick) of ovarian midsections were obtained with aid of a microtome and dried for 48 h at 37°C (Sampedro-Carrillo, 2022). Slides with ovarian slices were stored in plastic boxes to be used for all histological applications throughout the study.

Ovarian samples later excluded from the study were due to low quality of histological sections and material that did not fall within the scope of this article (perigonadal adipose tissue or uterine tissue). For this reason, in the results section of this article, the number of cases used will be mentioned for each technique.

As mentioned above, ovaries from mice had previously been embedded in paraffin, following the same protocol. For this study, new ovarian slices were obtained using the same procedures mentioned above regarding bitches’ ovaries.

### 2.3 Haematoxylin and eosin staining

Ovarian sections were dewaxed twice with xylol and hydrated with decreasing concentrations of ethanol (100%, 90% and 70%) and water. Hydrated sections were stained with Harris Haematoxylin (Thermo Scientific, 72,704) for 1 min, rinsed in tap water and then stained with alcoholic eosin (Sigma-Aldrich, HT1101128-4L) at 1% for 45 s. Dehydration was performed with increasing concentrations of ethanol (70%, 90% and 100%) followed by two incubations with xylol. Finally, tissue sections were mounted in Entellan^®^ and air-dried. Sequential application of the dyes to histologic sections results in nuclei being stained blue, and cytoplasm and extracellular matrix in varying degrees of pink (Feldman and Wolfe, 2014). Haematoxylin and eosin staining was used for ovarian morphology observation under a light microscope (Zeiss Axioskop 40) with a digital camera (Zeiss AxioCam 208 Color). Images from the longitudinal ovarian sections were captured using a digital slide scanner (Philips IntelliSite Pathology Scanner). Representative images were captured with both systems.

### 2.4 Follicle counting at ovarian midsection

Haematoxylin and eosin slides were used for follicle counting. The follicles were branded as primordial, primary, secondary, or antral. Follicles were classified as primordial or primary when oocytes were surrounded, respectively, by a single layer of squamous or cuboidal granulosa cells and secondary follicles when having more than one layer of granulosa cells with no visible antrum. Antral follicles were the ones that displayed areas of follicular fluid (antrum) or a single large antral space (Timóteo-Ferreira et al., 2021). The number of primordial and primary follicles at the ovarian midsection was obtained by calculating the count of an ovarian midsection representative of each animal by two independent researchers.

### 2.5 Sudan black B staining

Ovarian sections were dewaxed and hydrated as previously mentioned. Sections were then stained for 90 min with 1% Sudan Black B (Sigma-Aldrich, 199,664) in 70% ethanol (Evangelou and Gorgoulis, 2017). Excess staining was removed by rinsing slides in ethanol (70%) before a short incubation in phosphate-buffered saline (PBS) (M – 0.137 NaCl, 0.0027 KCl, 0.01 Na_2_HPO_4_, 0.0018 KH_2_PO_4_). Slices were mounted in 70% glycerol diluted in PBS and observed under a light microscope (Zeiss Axioskop 40) equipped with a digital camera (Zeiss AxioCam 208 Color) or mounted in Aquatex^®^ aqueous media and scanned using a digital slide scanner (Philips IntelliSite Pathology Scanner). Staining was quantified using the image J software by identification of the threshold cut point, with the blind intervention of the operator. Sudan Black B staining was used to identify lipofuscin present in multinucleated giant cells (Georgakopoulou et al., 2012).

### 2.6 IgG immunohistochemical staining

Ovarian sections were dewaxed twice with xylol and hydrated with decreasing concentrations of ethanol (100%, 90% and 70%, 5 min each) and water. Heat-induced antigen retrieval was performed. Tissue slices were submerged in boiling sodium citrate buffer (MB01501, nzytech) and kept in an oven at 70°C for 35 min. After cooling, endogenous peroxidase was blocked in 3% H_2_O_2_ in methanol for 30 min, at room temperature. Then, slides were rinsed three times with PBS with 0.1% Tween (PBS-T). To reduce background staining slides were treated with 5% bovine serum albumin in PBS-T, for 1 h at room temperature. Next, ovarian sections were incubated with the antibody against bitch or mice IgG labelled with horseradish peroxidase (HRP) (Table 1), overnight at 4°C. The following day, slides were washed three times in PBS-T and chromogenic detection was performed with 3,3′-diaminobenzidine, to which H_2_O_2_ in a proportion of 1:1,000 was added (Kim et al., 2016). Hematoxylin staining was used to counterstain the tissue. After dehydration, by going through a series of crescent percentages of ethanol (70%, 90% and 100%), ovarian slices were diaphanized with xylene, mounted in Entellan^®^ and scanned using a digital slide scanner (Philips IntelliSite Pathology Scanner). Representative images at mid-ovarian section were captured and staining quantified using the image J software by identification of the threshold cut point, with blind intervention of the operator.

**TABLE 1:** Description of the antibodies employed in the experimental procedures.

Antibodies			
Antigen	Code	Working dilution	Company
Goat anti-Canine IgG (HRP)	#A18763	1:25	Thermo fisher
Affini pure donkey anti-mouse (HRP)	715-035-150	1:20	Jackson immuno research

### 2.7 Western blotting for IgGs detection and quantification

Ovaries that had been stored at −80°C were homogenized, using a glass homogenizer (Heidolph), in ice-cold lysis buffer (10 mM NaCl, 5 mM EDTA, 50 mM Tris-HCl pH 7.5% and 1% SDS) containing proteases inhibitors (P8340; Sigma) and phosphatases inhibitors (P0044, P5726; Sigma). Afterwards, the lysates were sonicated and centrifuged at 1,500 g for 5 min. The pellet was rejected, and supernatants were stored at – 80°C for future experiments (Yang and Mahmood, 2012).

Equal amounts of protein were mixed with Laemmly sample buffer, heated at 65°C for 15 min, then at 95°C for 5 min and centrifuged at 15,000 g for 5 min. Samples were electrophoretically separated in a 12% SDS–polyacrylamide gel and electrotransfered to nitrocellulose membranes in Tris–glycine transfer buffer containing 20% methanol. Afterward, membranes were washed in distilled water, stained with Ponceau S and photographed in a ChemidocTM MP imaging system (BioRad^®^). Background signal was blocked with 2% bovine serum albumin in tris-buffered saline (0.1 M Tris, 2 mM Tris, 13.7 mM NaCl) with 0.1% of Tween 20 for 30 min at room temperature and subsequently membranes were incubated with the anti-mouse IgG HRP (Table 1) overnight at 4°C, with agitation. The following day, membranes were washed with tris-buffered saline with 0.1% of Tween 20 and revealed using a chemiluminescent agent (BioRad) for 5 min, at room temperature (Yang and Mahmood, 2012). The reaction was visualized in a ChemidocTM MP imaging system (BioRad^®^).

The Ponceau S staining was used for protein loading normalization and quantification was performed using Image Lab version 4.0.1 (BioRad^®^).

### 2.8 Statistical analysis

Statistical analyses were performed with GraphPad Prism 9.0.0 (GraphPad Software, California, United States). Spearman rank correlation coefficient was used to measure the correlation between the studied variables. The Kruskal-Wallis and Mann-Whitney-U tests were used for multiple comparisons between groups of animals with different ages. A *p* < 0.05 was assumed to denote statistical significance.

## 3 Results

### 3.1 Follicular reserve and ovarian aging

**FIGURE 1:**
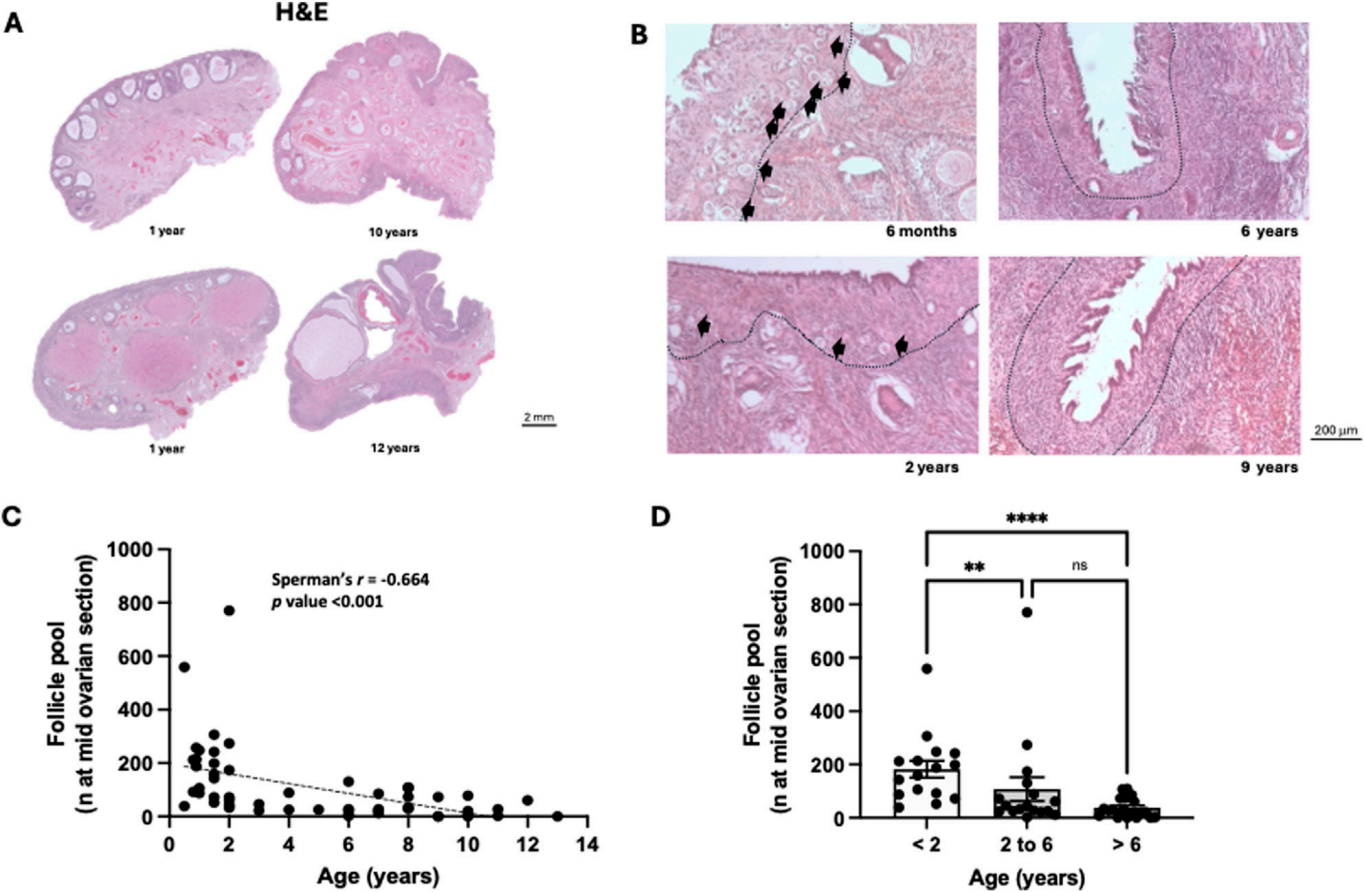
Bitch ovarian structure and follicle pool during ovarian aging. **(A)** Representative ovarian midsections from juvenile (less than 2 years) and reproductively aged animals (more than 6 years) stained with haematoxylin and eosin. **(B)** - Representative ovarian midsections of juvenile, mature and reproductively aged animals showing the distribution of primary and primordial follicles (black arrows) inserted in the ovarian cortex (dash line separates ovarian cortex from the medulla). **(C)** Negative correlation between the number of primordial/primary follicles at the mid-ovarian section and the age of the bitch (p < 0.001) (n = 53). **(D)** Average number of primordial/primary follicles per animal and age group. A significant decrease in follicle pool was observed in mature [2–6 years (n = 17)] and reproductively aged [more than 6 (n = 20) years] animals compared to juvenile animals [less than 2 years (n = 16)] (p < 0.05).

Ovarian midsections (n = 53) were observed under a light microscope (Figure 1A). In the cortex of ovaries from young bitches, the primordial and primary follicles were abundant, contrasting with those from aged bitches, where they were sporadic (Figure 1B). The follicular reserve at the mid-ovarian section, calculated by summing the number of primordial and primary follicles counted in a representative section of the ovary, was decreased with age (Figure 1C). The Spearman correlation test revealed a strong, negative, and significant association between follicular reserve and age (*r* = −0.644; *p* < 0.001) (Figure 1C).

Subsequently, the sample was organized into age groups as follows: juvenile group - animals under 2 years old; mature group - animals between 2 and 6 years old inclusive; and reproductively aged group - animals over 6 years old (Figure 1D). Aging significantly affected follicle reserve (Kruskal-Walli’s test: *p* < 0.001), with a significant decrease (*p* < 0.01) between the juvenile and mature groups and between the juvenile and reproductively aged groups. No significant differences were observed between the mature and reproductively aged groups (Figure 1D).

### 3.2 Hallmarks of ovarian aging

**FIGURE 2:**
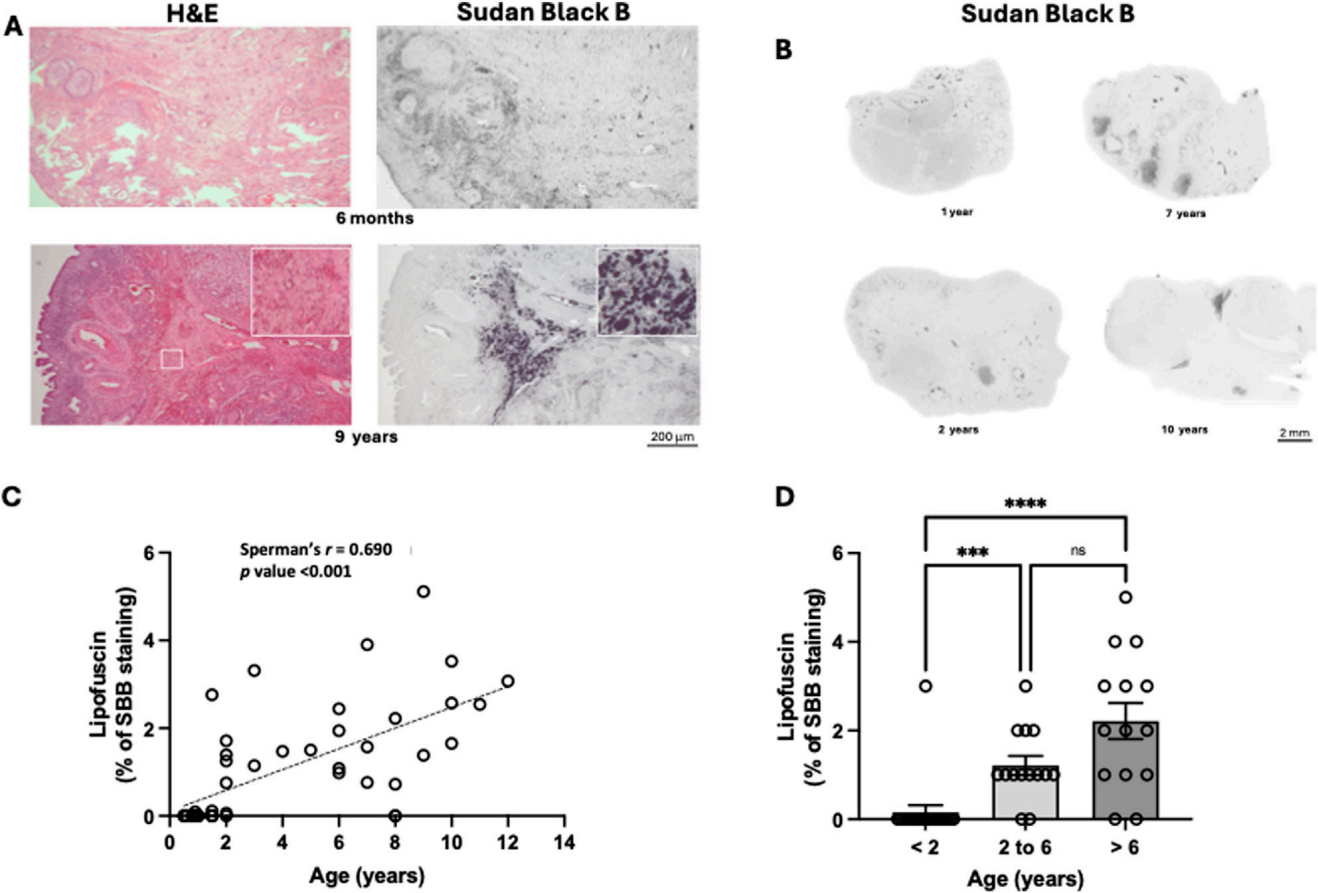
Identification of multinucleated giant cells during ovarian aging, in the bitch. **(A)** — Representative image showing multinucleated giant cells only in the ovary of a reproductively aged bitch. Multinucleated giant cells have a light dark brown cytoplasm when stained with haematoxylin and eosin (insert in figure from 9-year-old bitch) and lipofuscin accumulation (positivity for Sudan black B). **(B)** Representative ovarian midsections stained with Sudan black B. **(C)** Positive correlation between the lipofuscin deposition at the mid-ovarian section and the age of the bitch (p < 0.001) (n = 47). **(D)** Average values of lipofuscin deposition per age group. A significant increase in lipofuscin deposition was observed in mature [2–6 years (n = 14)] and reproductively aged [more than 6 years (n = 14)] animals compared to juvenile animals [less than 2 years (n = 19)] (p < 0.05).

The ovarian accumulation of multinucleated giant cells, with a pale cytoplasm and lipofuscin deposits, was also investigated in this study (n = 47) (Figure 2A). These cells were virtually absent in juvenile animals (with less than 2 years) (Figures 2A,B). It was found, by quantification of lipofuscin deposits, that during reproductive aging there is an increase in ovarian multinucleated giant cells, especially in the medulla and in areas surrounding secondary follicles and *corpus luteum* (Figure 2A). A strong, positive, and significant correlation was observed between the age of the bitches and the lipofuscin deposits (*r* = 0.713; *p* < 0.001) (Figure 2C).

Ovarian lipofuscin deposition increased with the age of the animals (Kruskal-Walli’s test: *p* < 0.001). A significant increase (*p* < 0.001) was observed between the juvenile and mature groups and the juvenile and reproductively aged groups (Figure 2D). No significant differences were observed between the mature and reproductively aged groups. It is important to note that in young animals (under 2 years), this type of staining is practically absent, indicating that the presence of multinucleated giant cells with lipofuscin accumulation occurs in early adulthood.

### 3.3 IgG and ovarian aging

**FIGURE 3:**
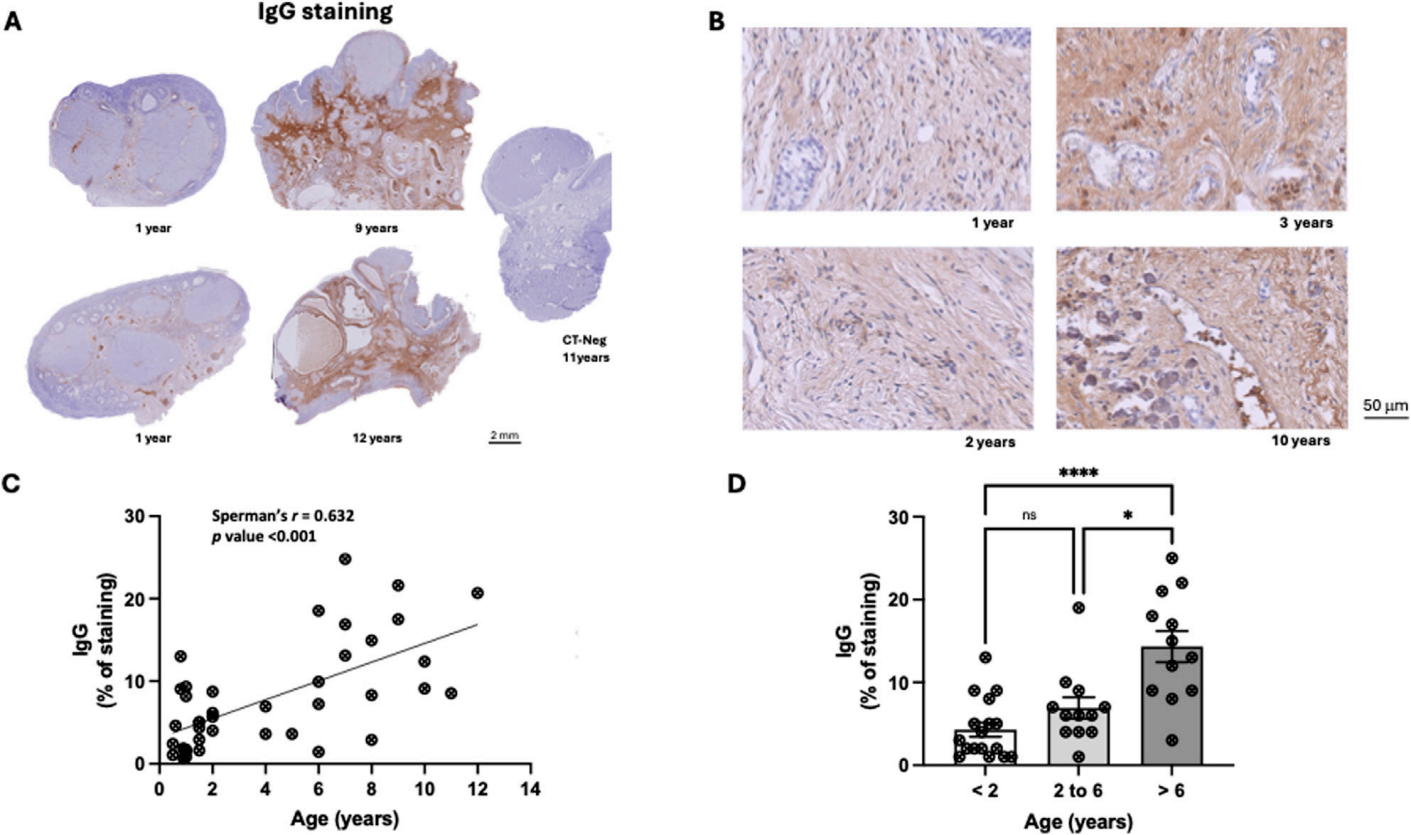
Immunoglobulins accumulation during ovarian aging in the bitch. **(A)** Representative immunohistochemistry for IgG at ovarian midsections from juvenile and reproductively aged animals. **(B)** Representative ovarian midsections of juvenile and reproductively aged animals showing IgG accumulation. **(C)** Positive correlation between the IgG accumulation at the mid-ovarian section and the age of the bitch (p < 0.001) (n = 41). **(D)** Average values of IgG accumulation per age group. A significant increase in IgG accumulation was observed in reproductively aged [more than 6 years (n = 12)] animals compared to juvenile [less than 2 years (n = 17)]) and mature [2–6 years (n = 12)] animals (p < 0.05).

Since there were no data on IgG abundance in the ovary and its correlation with reproductive aging this was also evaluated in the ovarian samples of bitches (n = 41). IgG were detected in the ovaries of the bitches (Figure 3A), including younger animals, where IgG immunostaining appeared diffuse in the ovarian medulla (Figures 3A,B). The deposition of IgG increased with age, with the immunostaining pattern predominantly remaining diffuse and more intense in the ovarian medulla. Occasionally, cytoplasmic or nuclear staining was observed within follicular or medullary cells, as well as within blood vessels (Figure 3B). The Spearman correlation analysis showed a moderate, positive, and significant association between the age of the bitches and the abundance of IgG (*r* = 0.598; *p* < 0.001) (Figure 3C).

Significant differences in IgG accumulation between groups were observed (Kruskal-Walli’s test: *p* < 0.01), with a significant increase (*p* < 0.05) observed between the juvenile and reproductively aged groups, and the mature and reproductively aged groups (Figure 3D). No significant differences were observed between the juvenile and mature groups.

### 3.4 Follicular pool, lipofuscin deposits and IgG accumulation

**FIGURE 4:**
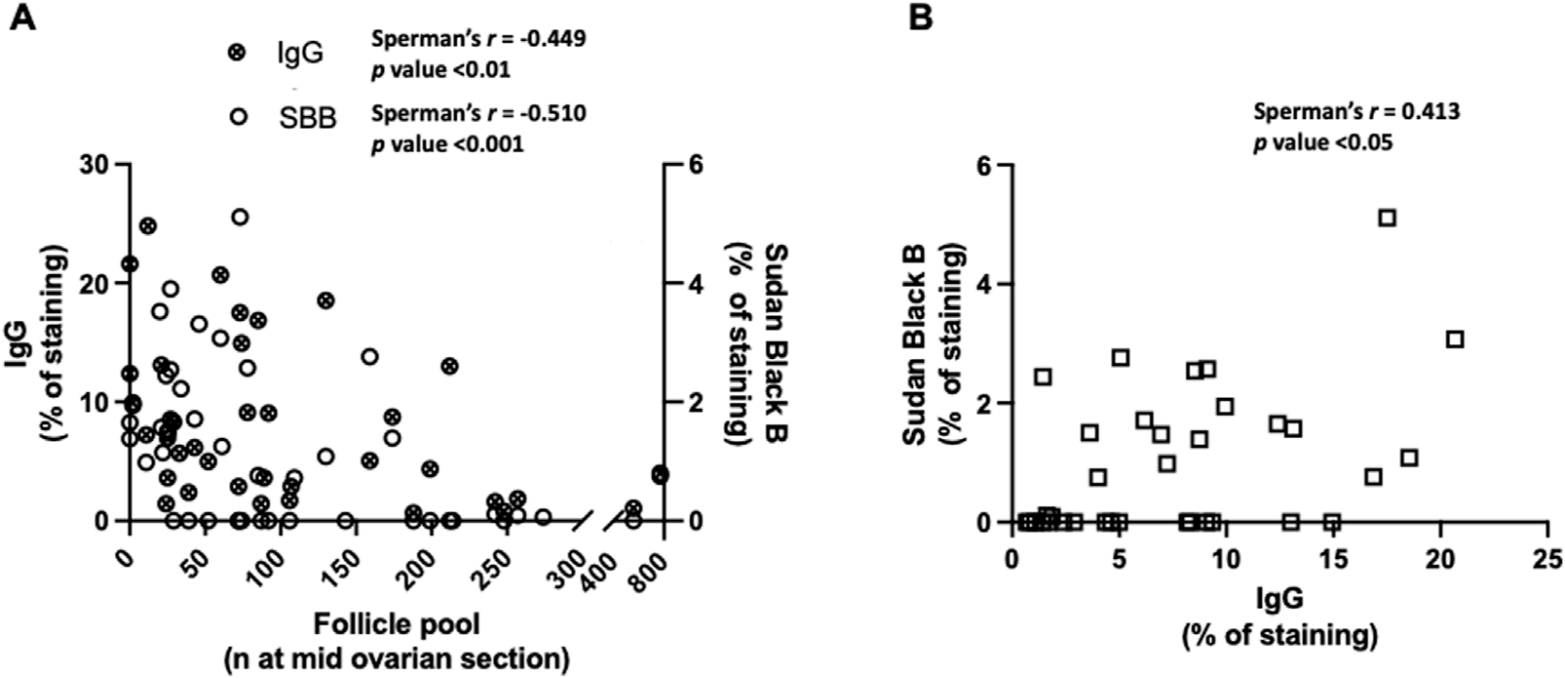
Follicle decline and correlations with markers of ovarian aging in the bitch. **(A)** Negative correlations between follicle pool and the IgG accumulation (n = 37) and follicle pool and lipofuscin deposition (n = 46). **(B)** Positive correlation between the IgG and lipofuscin (n = 35) (*p* < 0.001).

Additionally, it was also evaluated the correlation between follicle pool, lipofuscin deposits and IgG accumulation in the ovaries of the bitches. As depicted in Figure 4A both lipofuscin deposition and IgG accumulation correlate negatively with follicle pool. However, despite the existence of a positive correlation between lipofuscin deposition and IgG accumulation it is possible to infer by observation of Figure 4B that the percentage of IgG staining starts to increase before lipofuscin accumulation.

### 3.5 IgG and ROS

**FIGURE 5:**
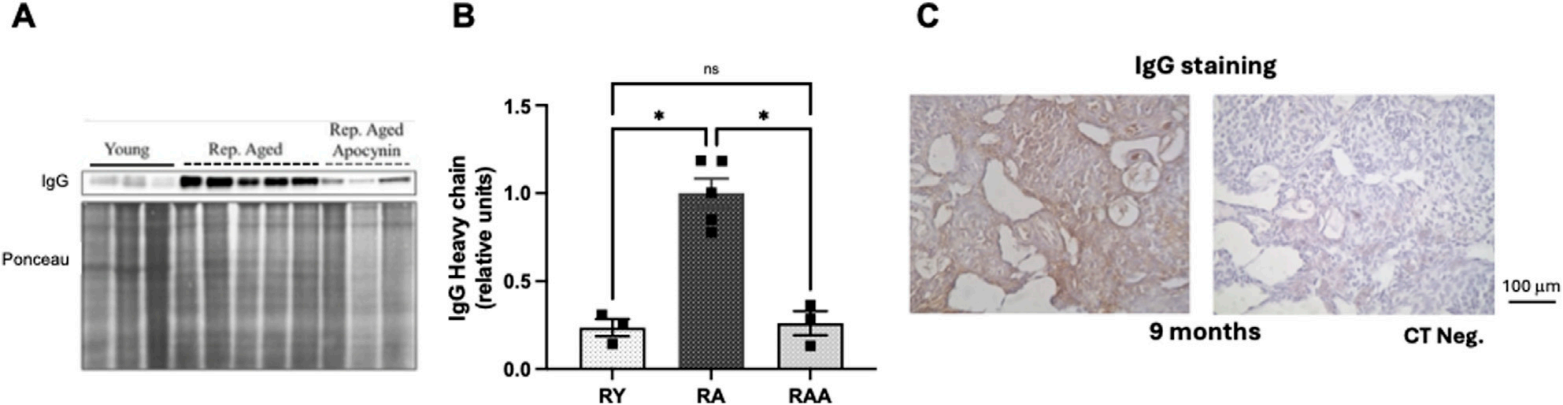
Role of oxidative stress in ovarian IgG accumulation during mice reproductive aging. **(A)** Western blotting image showing IgG heavy chain bands and the corresponding Ponceau (loading control). **(B)** Average values of IgG abundance per age group. A significant increase in ovarian IgG was observed in reproductively aged [9 months-old (n = 5)] animals compared to young [3 months-old (n = 3)] (*p* < 0.05). Apocynin treatment reverted IgG accumulation in reproductively aged animals (n = 3) (*p* < 0.05). **(C)** Representative immunohistochemistry for IgG at ovarian midsections of reproductively aged mice.

Next, it was evaluated whether IgG also accumulated in the ovaries of reproductively aged (9 months-old) mice. Using ovarian tissue homogenates from mice it was possible to demonstrate that, like what is observed in the bitch, there is a significant increase in ovarian IgG abundance in the group of reproductively aged animals (n = 5), in comparison to the young group (3 months-old) (n = 3) (Figures 5A,B). As we had a group of reproductively aged animals treated with the antioxidant apocynin (n = 3) it was possible to observe that it significantly decreased immunoglobulins in the ovaries of reproductively aged mice (Figures 5A,B). Additionally, immunohistochemistry was also performed in ovaries from aged mice allowing us to observe that there is a similar pattern of IgG staining, when compared to the bitch (predominant staining in the stoma) (Figure 5C).

## 4 Discussion

The present study provides a comprehensive analysis of ovarian aging in the bitch. It shows that ovarian aging is accompanied by a decrease in the follicle pool, and that this decrease is negatively correlated with lipofuscin deposition. Importantly, although these features have been observed in other species, to our knowledge, this is the first time that such changes have been described in the canine ovary. Moreover, our study provides a comprehensive age-range analysis, including animals from less than 1 year up to 13 years of age, thus offering a continuous and progressive evaluation of ovarian aging. It also reports, for the first time, an age-related increase in ovarian accumulation of IgG, that is normalized by treatment with an antioxidant molecule. These findings favor IgG and ROS role as part of a coordinated mechanism driving ovarian aging.

As anticipated, the number of primordial and primary follicles at the mid-ovarian sections showed a clear age-dependent decline, which aligns with the established knowledge regarding ovarian physiology in reproductive aging. The continuous follicle recruitment, proceeded by their development, atresia of underdeveloped follicles and ovulation of the dominants, underlies the observed age-related decrease (Hansen et al., 2008; Pelosi et al., 2015). In addition, apart from the age-related loss of follicle number, other studies emphasized that, perhaps more importantly, age-related fertility reduction is associated with an increasing number of poor-quality oocytes (Bao et al., 2024; Moghadam et al., 2022; Prasad et al., 2016; Tarín et al., 2002), for reasons that remain unclear.

Interestingly, during aging, the ovarian stroma is colonized by a unique population of multinucleated giant cells (Briley et al., 2016; Shen et al., 2024), positive for the macrophage marker f4/80, displaying lipofuscin inclusions and iron deposition (Asano, 2012; Urzua et al., 2018).

Macrophages reflect the continued homeostatic effect of clearing dead cells or cell debris by phagocytosis, a frequent event in the functional ovary, antedating the significant loss of follicles (Ansere et al., 2021). Moreover, the enlarged area that they occupy in the ovaries of older bitches parallels the previous observation in the ovaries of mice, ovines and caprines (Briley et al., 2016; Montenegro et al., 2023; Timóteo-Ferreira et al., 2019), favoring the existence of a peculiar mammalian event in the aged ovary.

In the present study, lipofuscin was nearly absent in the ovaries of bitches up to 2 years of age. In contrast, its significant presence in ovarian multinucleated giant cells of older bitches suggests a progressive accumulation of ill-digested phagocytosed material over time, stresses its recognition as a hallmark of aging and highlights the involvement of these immune cells in the ovarian aging process itself.

Beyond their homeostatic effect, macrophages secrete pro-inflammatory cytokines, exacerbate oxidative stress and oxidative damage, and promote tissue fibrosis (Ansere et al., 2021; McNally and Anderson, 2011; Shen et al., 2024; Wynn and Barron, 2010). These roles in the aged ovary were previously approached when we evidenced correlation with inflammatory responses (Briley et al., 2016; Timóteo-Ferreira et al., 2019).

The presence of IgG in the ovaries of young animals was an unexpected finding, as this is an age typically associated with optimal bitch fertility. As the presence of IgG in the ovaries was observed in the absence of multinucleated giant cells or visible lipofuscin accumulation, it suggests that IgG may precede these other hallmarks of ovarian aging, even if it did not reach significance.

The observed increase in ovarian IgG levels could result from enhanced vascularization, leading to greater blood content within the tissue. However, Mu L and co-workers (Mu et al., 2025) demonstrated a significant decline in ovarian blood vessel density and angiogenesis already in middle-aged mice. This vascular reduction was linked to reduced ovarian function and fertility decline. Authors show that, rather than an increase, there is a notable decrease in ovarian vascularization with age. It is unlikely that the elevated IgG levels observed in our study are due to increased blood content from enhanced vascularization. The distribution pattern of IgG further reinforces that the increased presence is not due to enhanced vascularization as staining is more abundant in the stroma.

Despite our data not establishing a causal or temporal hierarchy, these findings raise the possibility that IgG presence could represent an early immune-modulatory event in the aging process, which deserves further investigation. Local IgG could act as a signaling molecule for macrophage recruitment. In fact, in basal conditions, macrophages endocytose and recycle large amounts of IgG through its pivotal membrane bound FcRN receptor (Challa et al., 2019) whose activity prevents IgG rapid catabolism (Kim et al., 2007), thus exerting a IgG sparing role.

Irrespective of whether IgG acts as a signaling molecule or a chemoattractant for macrophage recruitment, there is consistent evidence supporting its involvement in the aging process. Landry et al. (2022) reported that aging alters the immune landscape of the ovary, characterized by an increase in the proportions of B, T and natural killer T cells, among others. B cells could be the source of ovarian IgG, but further studies will be necessary to address this issue. Additionally, a recent study performed in adipose tissue provides new insights into the role of IgG in aging. Authors demonstrate that IgG accumulates in several tissues during aging and that, in the adipose white tissue (WAT), IgG induces tissue fibrosis via activation of macrophages (Yu et al., 2024).

In the ovary, the early presence of IgG could be responsible for the initiation of an immune response with similar consequences. The early presence of IgG in the ovaries, before the onset of significant lipofuscin accumulation, opens new avenues for understanding the mechanisms driving ovarian aging. Similarly to the perspective regarding the WAT (Yu et al., 2024), it raises the possibility that interventions targeting to decrease ovarian IgG have the potential to reduce the recruitment of macrophages and local inflammatory or stressful conditions and delay the aging of the ovary. As the canine ovaries used in this study were obtained from animals undergoing elective ovariohysterectomy at veterinary clinics it was not possible to perform interventional experiments, such as antioxidant administration aiming to evaluate whether it was possible to reduce IgG presence in the ovaries, by reducing the levels of reactive oxygen species. To overcome this limitation ovarian tissue from reproductively aged mice (9 months old) previously treated with apocynin was used.

Work from our group with those animals demonstrated that the age-associated ovarian increase in inflammation and fibrosis could be mitigated with the use of the antioxidant apocynin. Apocynin was able to reduce ovarian oxidative damage, markers of inflammation and fibrosis in reproductively aged mice (9 months old) (Timóteo-Ferreira et al., 2019). The results from the present study, provide evidence that, in addition, it can reverse the accumulation of IgG in the ovaries of reproductively aged animals. The exact mechanism by which apocynin reduces IgG in the ovaries remains unknown. Yu et al. (2024) reported that IgG accumulation in WAT during aging, leads to macrophage activation via Ras signaling and subsequent fibrosis through the TGF-β/SMAD pathway. The authors demonstrated that caloric restriction reduces IgG accumulation in WAT, while replenishing IgG opposes/abolishes the metabolic benefits of the intervention. Furthermore, mice lacking B cells were protected from aging-associated WAT fibrosis, inflammation, and insulin resistance unless exposed to IgG. Altogether, it is thus suggested that IgG accumulation can initiate immune responses leading to tissue inflammation and fibrosis, contributing to functional decline during aging.

Although our study did not directly test this mechanism in ovarian tissue, the parallels in immune-mediated aging processes across different tissues support that IgG accumulation may similarly impact ovarian aging. Its elucidation will provide insights for the development of targeted therapies to delay ovarian aging, with an impact in human and non-human mammalian reproductive medicine.

In conclusion, this study confirms that the follicle pool diminishes, and lipofuscin deposition increases with the age of the bitches. More importantly, it identifies an early deposition of IgG in the ovaries which fits within a broader context of immune changes in ovarian aging and contributes to an upsurge in the understanding of ovarian aging and potential strategies to mitigate it.

## Data Availability

The raw data supporting the conclusions of this article will be made available by the authors, without undue reservation.
