# Supplementary material for: Ovarian ROS-dependent IgG accumulation precedes lipofuscin deposition and follicular decline: comparative insights from the bitch and mouse models of ovarian aging
Source: Front Aging. 2025 May 30;6:1567909. doi: 10.3389/fragi.2025.1567909 (PMC12162967; doi:10.3389/fragi.2025.1567909)
Supplement: Supplementary file 1 [file Presentation1.pptx]

## Slide 1
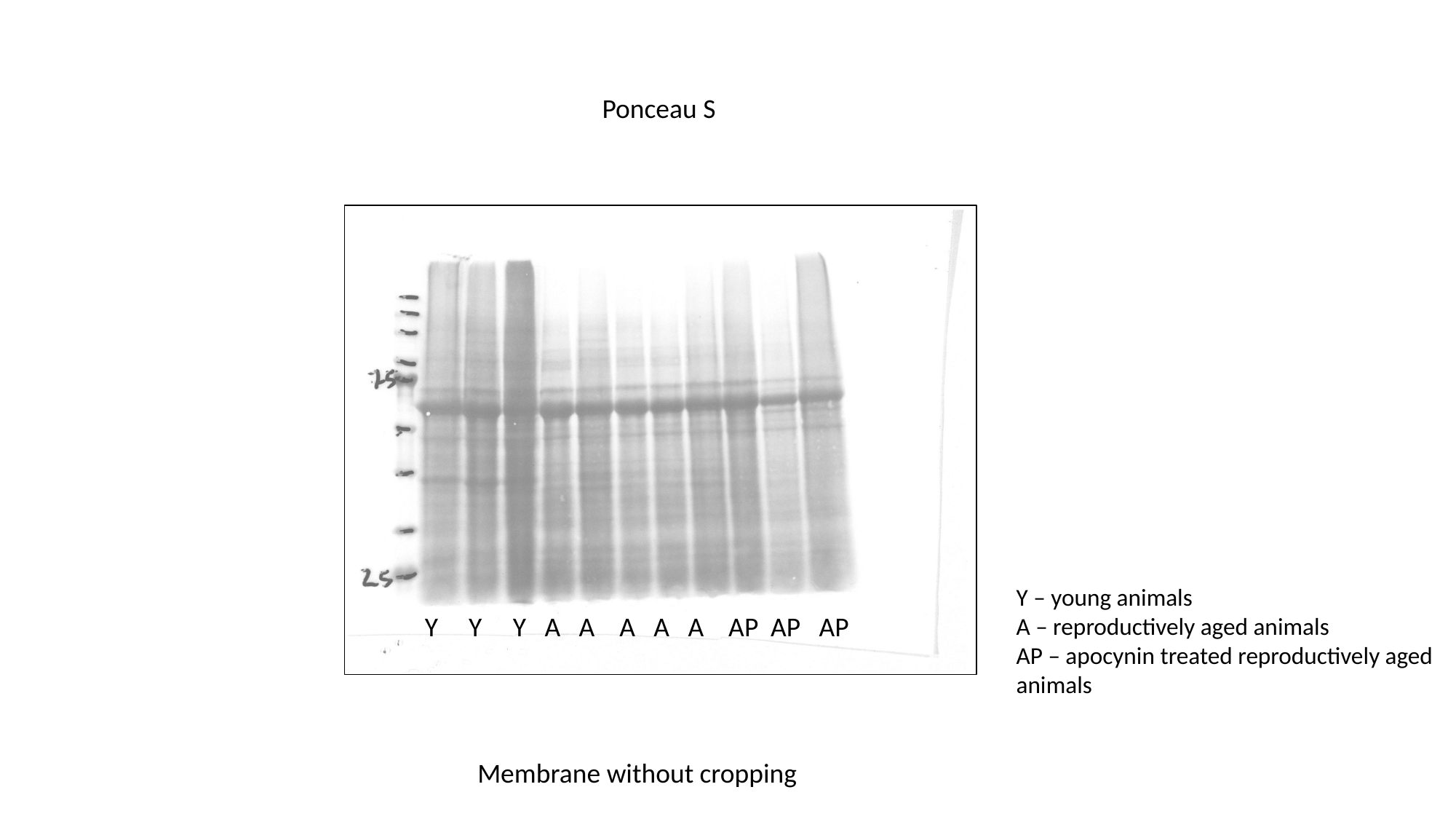

Ponceau S
Y – young animals
A – reproductively aged animals
AP – apocynin treated reproductively aged animals
Y Y Y A A A A A AP AP AP
Membrane without cropping

## Slide 2
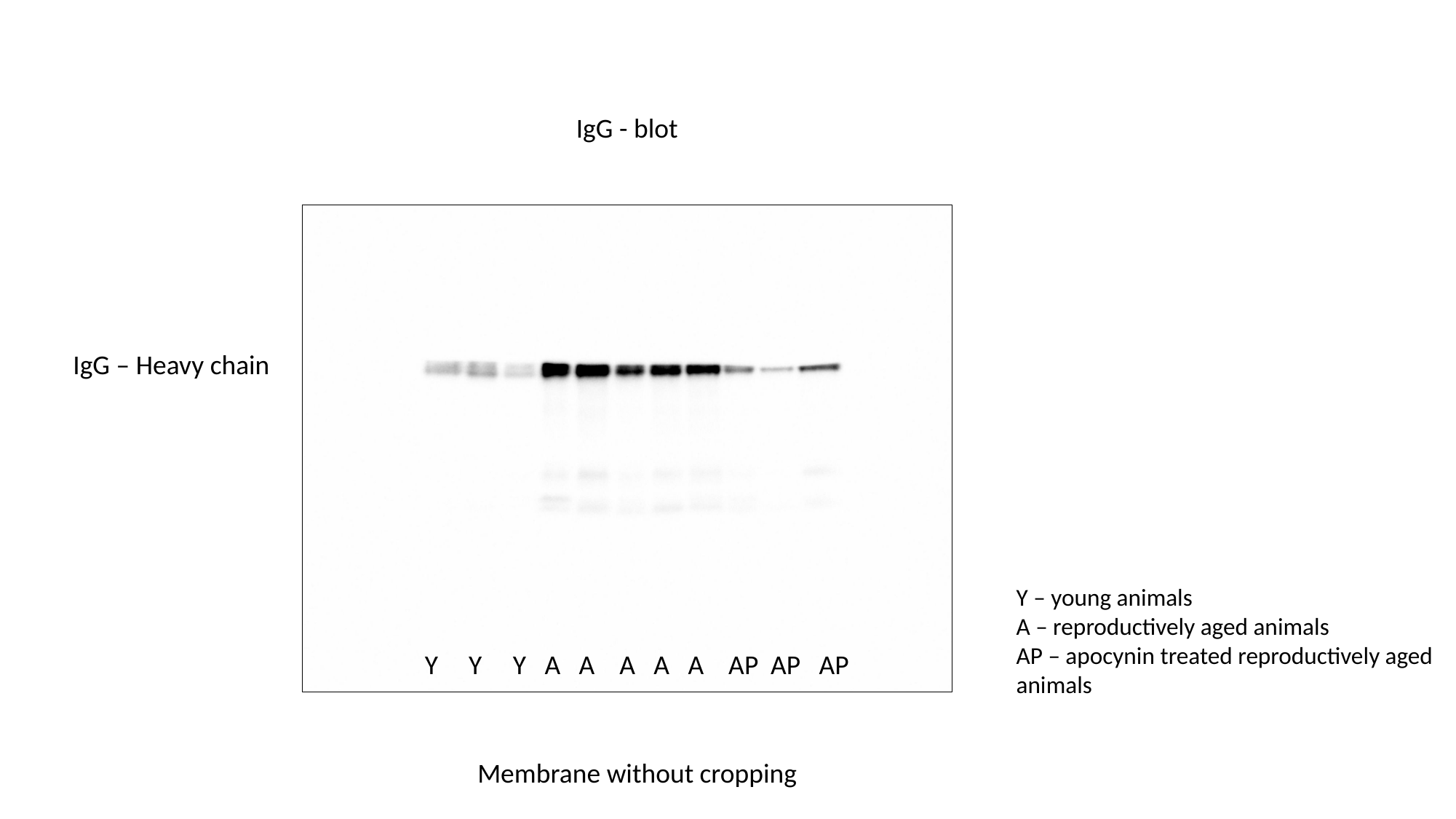

IgG - blot
IgG – Heavy chain
Y – young animals
A – reproductively aged animals
AP – apocynin treated reproductively aged animals
Y Y Y A A A A A AP AP AP
Membrane without cropping
